# Cutaneous metastatic carcinoma from gastric adenocarcinoma: Atypical facial and neck eczematous presentation

**DOI:** 10.1016/j.jdcr.2026.03.032

**Published:** 2026-03-20

**Authors:** Scott Kuan-Wen Wang, Dean George, Goran Micevic

**Affiliations:** a Department of Pathology and Laboratory Medicine, Hartford Hospital, Hartford, Connecticut; b Hartford Healthcare Cancer Institute, Hartford Hospital, Hartford, Connecticut; c Department of Dermatology, Yale School of Medicine, New Haven, Connecticut; d Department of Pathology, Yale School of Medicine, New Haven, Connecticut

**Keywords:** carcinoma erysipeloides, eczematous, gastric adenocarcinoma

## Introduction

Cutaneous metastases represent an uncommon clinical manifestation of internal malignancies, occurring in approximately 0.7% to 9% of all cancer patients.^1^^,^^2^ Among the various cutaneous metastatic patterns, carcinoma erysipeloides (CE) is a rare and distinctive subtype characterized by tumor cell infiltration of dermal lymphatic vessels, producing erythematous, warm, and indurated plaques that mimic infectious processes such as erysipelas or cellulitis.^2^ Because of this resemblance, CE is frequently misdiagnosed in its early stages, leading to delayed identification of the underlying malignancy. Here we discuss a patient with an unusual clinical presentation of carcinoma erysipeloides.

## Case report

**Fig 1:**
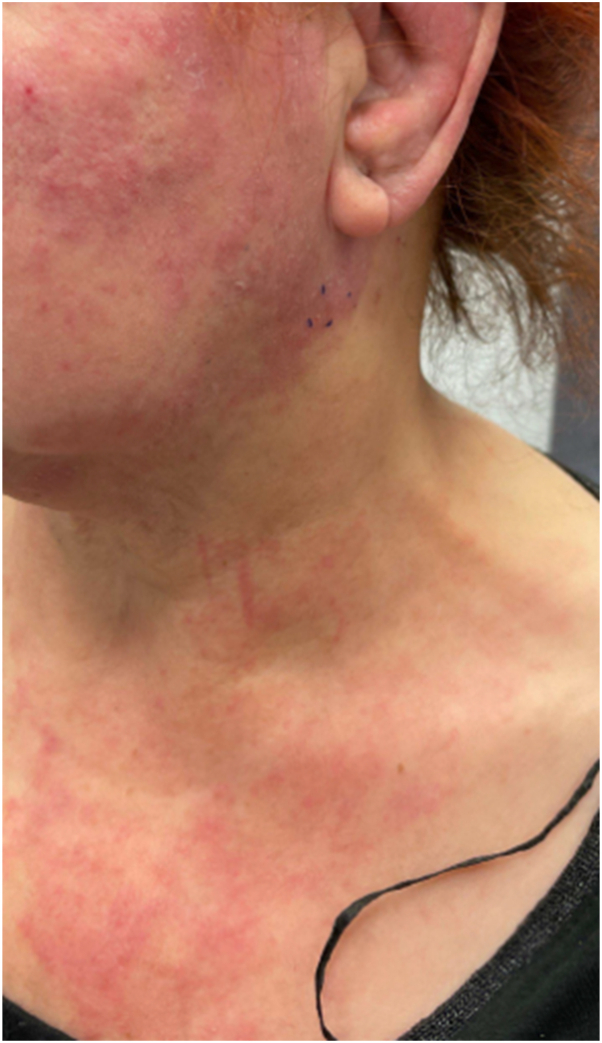
Clinical presentation. *Pink* patches and papules coalescing into thin plaques with focal scale affecting the cheek, neck, and chest.

**Fig 2:**
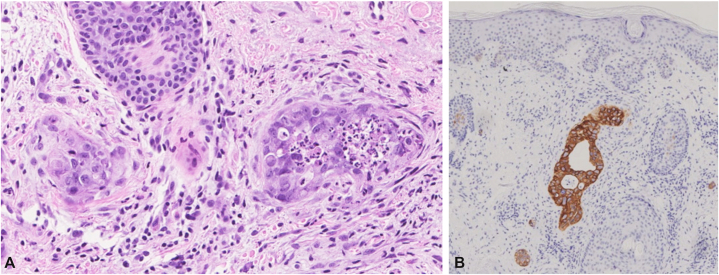
Histopathologic finding. **A,** Islands of atypical cells are present in the lumen of small vessels of the superficial dermis (hematoxylin and eosin, 200×). **B,** Atypical cells are positive for CK7 (40×).

A woman in her 60s without significant medical history presented to the general practitioner with a pruritic rash extending from her left ear down to her neck and abdominal discomfort for a few months. She was referred to the dermatologist due to the rash had been refractory to topical steroid treatment. Cutaneous examination revealed multiple erythematous-pink patches and papules coalescing into thin plaques with focal scale distributed across her left facial region, neck, and anterior chest wall (Fig 1) with mild induration but no tenderness. Given the atypical clinical presentation and lack of response to conventional therapy, a skin biopsy at the edge of erythematous rash was performed. Histopathological analysis demonstrated clusters of atypical epithelial cells within the lumina of superficial dermal lymphatic vessels (Fig 2, *A*). Immunohistochemical staining revealed strong positivity for cytokeratin 7 (Fig 2, *B*) and absence of cytokeratin 20 expression. D2-40 immunohistochemical staining was not performed due to the specimen limitation. Based on the morphology and immunohistochemical profiles suggestive of a differential diagnosis of primary cutaneous apocrine carcinoma, metastatic carcinoma from breast, lung, endometrium, ovary or upper gastrointestinal tract origin.

Concurrent imaging via abdominal computed tomography identified nodularity of the omentum, raising suspicion for peritoneal carcinomatosis. Further endoscopic evaluation revealed a 6-mm, cratered gastric ulcer in the gastric body, without active bleeding. Histopathological examination of this gastric lesion confirmed gastric adenocarcinoma with prominent signet ring cell morphology. The morphology and immunophenotypic profile of the gastric malignancy matched that of the cutaneous lesion, supporting the diagnosis of cutaneous metastasis. Metastatic dissemination to the omentum was also established, highlighting advanced-stage disease.

Based on these clinical and histopathological findings, a diagnosis of CE secondary to gastric adenocarcinoma was established. The patient was subsequently managed with systemic chemotherapy combined with an immune checkpoint inhibitor. Continuous follow-up over 1 year demonstrated persistent disease, albeit with slight clinical improvement and partial response to therapy.

## Discussion

Carcinoma erysipeloides represents a distinctive yet uncommon manifestation of cutaneous metastasis, characterized by tumor emboli in dermal lymphatics resulting in inflammatory skin changes that closely mimic infectious cellulitis or erysipelas.^3^ While breast carcinoma remains the most frequent malignancy associated with CE, gastrointestinal adenocarcinomas are considerably rarer primary tumors.^4^^,^^5^ Specifically, cutaneous metastasis arising from gastric cancer is exceedingly rare, with fewer than 30 cases documented in the literature; notably, approximately one-third of these cases display a signet ring cell morphology.^5^^,^^6^ The rarity and variability in clinical presentations contribute to diagnostic challenges, often resulting in delayed recognition and suboptimal initial management.

The cutaneous manifestation of internal malignancy as CE is generally associated with advanced disease and poor prognosis, underscoring the critical importance of early clinical suspicion and timely histopathological evaluation. This particular case emphasizes the atypical involvement of the face and neck, regions rarely documented as primary sites for CE originating from gastric adenocarcinoma. Moreover, the unusual clinical presentation of pruritus, and eczematous rather than nodular appearance^7^ further complicated the diagnostic process. Early recognition of such atypical metastatic patterns through comprehensive clinical evaluation and heightened pathological vigilance is essential to optimizing patient outcomes. Repeated sampling at multiple dermal levels has been suggested for suspected CE due to the absence of dermal lymphatic invasion.^8^

In conclusion, this case exemplifies carcinoma erysipeloides as an uncommon and diagnostically challenging cutaneous metastatic manifestation of gastric adenocarcinoma, highlighting the importance of maintaining a high index of clinical suspicion for metastatic disease, particularly when encountering treatment-resistant inflammatory dermatoses.

## Conflicts of interest

None disclosed.
